# Genome-wide *In Silico* Analysis, Characterization and Identification of Microsatellites in Spodoptera littoralis Multiple nucleopolyhedrovirus (SpliMNPV)

**DOI:** 10.1038/srep33741

**Published:** 2016-09-21

**Authors:** Mohamed A. M. Atia, Gamal H. Osman, Wael H. Elmenofy

**Affiliations:** 1 Genome Mapping Dept., Agricultural Genetic Engineering Research Institute (AGERI), ARC, Giza, 12619, Egypt; 2 Biology Department, Faculty of Applied Sciences, Umm Al-Qura University, Makkah, 21955, PO Box 715, Saudi Arabia; 3 Microbial Genetics Dept., Agricultural Genetic Engineering Research Institute (AGERI), ARC, Giza, 12619, Egypt

## Abstract

In this study, we undertook a survey to analyze the distribution and frequency of microsatellites or Simple Sequence Repeats (SSRs) in Spodoptera littoralis multiple nucleopolyhedrovirus (SpliMNPV) genome (isolate AN–1956). Out of the 55 microsatellite motifs, identified in the SpliMNPV-AN1956 genome using *in silico* analysis (inclusive of mono-, di-, tri- and hexa-nucleotide repeats), 39 were found to be distributed within coding regions (cSSRs), whereas 16 were observed to lie within intergenic or noncoding regions. Among the 39 motifs located in coding regions, 21 were located in annotated functional genes whilst 18 were identified in unknown functional genes (hypothetical proteins). Among the identified motifs, trinucleotide (80%) repeats were found to be the most abundant followed by dinucleotide (13%), mononucleotide (5%) and hexanucleotide (2%) repeats. The 39 motifs located within coding regions were further validated *in vitro* by using PCR analysis, while the 21 motifs located within known functional genes (15 genes) were characterized using nucleotide sequencing. A comparison of the sequence analysis data of the 21 sequenced cSSRs with the published sequences is presented. Finally, the developed SSR markers of the 39 motifs were further mapped/localized onto the SpliMNPV-AN1956 genome. In conclusion, the SSR markers specific to SpliMNPV, developed in this study, could be a useful tool for the identification of isolates and analysis of genetic diversity and viral evolutionary status.

Baculoviruses, the most common type of insect specific viruses, are extremely diverse with interesting applications and a wide host range (about 600 species of insects worldwide). They are enveloped viruses with a circular double-strand DNA genome that ranges in size from 80 to 200 kb^1^. Baculoviruses are popularly regarded as pathogens that are specific for invertebrates especially insects of the order Lepidoptera, Hymenoptera and Diptera^2^. In this respect, baculoviruses have garnered a significant amount of attention as potential agents for biological control of pests belonging to the abovementioned orders. As an added advantage, increasing our insight into baculovirus molecular biology has enabled us to optimally utilize viruses as vectors for the expression of foreign proteins inside insect cells^3^. As an aid to further augment our understanding of the molecular genetics of these viruses, several baculoviral genomes have been sequenced in the last two decades. The first completely sequenced baculoviral genome belonging to the Autographa californica Multiple Nucleopolyhedrovirus (AcMNPV) was reported by Ayres *et al*.^4^. To the best of our knowledge, there are 73 fully sequenced baculoviral genomes available in GenBank (41 genomes of the Alpha-baculovirus genus, 13 of the Beta-baculovirus, 3 of the Gamma*-*baculovirus and one of the Delta*-*baculovirus). The number of fully sequenced baculoviral genomes available to us are infinitesimally low when compared to the number of species that exist in nature (about 600 species)^5^. However, with the development of advanced molecular biology based techniques such as gene cloning and sequencing, DNA restriction analysis and molecular phylogeny, several new and highly useful tools for gene and genome characterization are now accessible to us. The major impediment to the absence of clarity regarding the real diversity of baculoviruses is because of the absence of reliable system for virus identification^5^. Simple sequence repeats (SSRs), also known as microsatellites, refer to mono-, di-, tri-, tetra-, penta- and hexanucleotide sequence units that are repeated in tandem in a genome^6^,^7^. Microsatellites are widely regarded as the most variable type of DNA sequence within the viral genome. SSRs are found in a variety of genomic regions including the 3′ and 5′ untranslated regions as well as exons and introns (protein-coding and non-coding regions)^8^,^9^,^10^. For this reason, SSRs are speculated to play a variety of diverse roles in the eukaryotic, prokaryotic and viral genomes. In spite of their hypermutable nature, SSRs have been widely used as markers for a variety of studies such as genome mapping, ecology and evolutionary genetics. It is also well known that the inherent instability of microsatellites plays a crucial role in the development of frame shift mutations that encode phenotypic changes and confer an adaptive advantage for the evolution of certain mutated viral strains^11^. Despite the growing number of completely sequenced viral genomes submitted to the public database, little attention has been paid towards surveying SSRs at the genome level for viruses in general and baculoviral genomes in particular. In spite of their abundance and functional relevance in viral genomes, the distribution pattern of microsatellites remains to be fully elucidated^7^. As a result of this, determining microsatellites distribution in baculoviruses has become crucial for understanding the evolution of baculoviral genomes. *Spodoptera littoralis*, the Egyptian cotton leafworm, causes significant damage to a wide range of economically important crops in Africa, southern Europe and in Middle East^12^. Recently, full genome sequencing of the *S. littoralis* multiple nucleopolyhedrovirus (SpliMNPV) revealed that the viral genome is 137,998 bp in size and is composed of 132 open reading frames and 15 homologous repeat regions^13^. In the current study, as an attempt to develop specific microsatellites markers to SpliMNPV, we present results from a genome-wide *in silico* analysis, characterization and identification of microsatellites distribution within the SpliMNPV genome. We propose that the results have the potential to expand our understanding of virus diversity, evolution and isolate identification.

## Results

### Distribution of SSRs in SpliMNPV Genome

**Figure 1:**
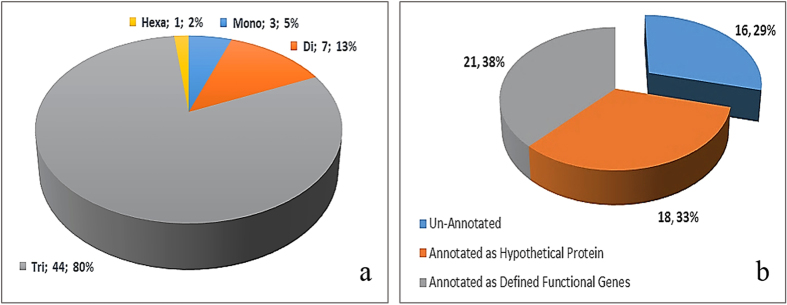
(**a**) Types of SSR motifs, number of motifs for each type and their frequency in SpliMNPV genome. (**b**) Distribution of SSR motifs on SpliMNPV genome components and their frequency.

In the present study, we analyzed the distribution of perfect SSRs (1–6 bp long) within the *S. littoralis* nucleopolyhedrovirus (SpliMNPV-AN1956) genome. Our attempts were successful in identifying 55 different SSR motifs (mono-, di-, tri- and hexa-nucleotide repeats) distributed within the SpliMNPV genome sequence. Interestingly, there were no tetra- and penta-nucleotide repeats observed in the SpliMNPV genome. Among the identified SSR motifs, trinucleotides (44 motifs; 80%) were the most common type of repeats followed by dinucleotides (7 motifs; 13%), mononucleotides (3 motifs; 5%) and lastly, the hexa-nucleotides (1 motif; 2%) motifs (Fig. 1a). Sixteen (29%) microsatellite motifs were found to be distributed within intergenic or noncoding regions, while 39 (71%) were present in Open Reading Frames (ORFs or coding regions; cSSRs). Of these 39 cSSR motifs, 21 were localized within defined functional genes, while 18 were present within Coding DNA Sequence (CDS) regions annotated as hypothetical proteins (with unknown function). As some genes were found to harbor more than one microsatellite repeat region, the 21 different cSSR motifs were found to be localized within 15 defined functional genes (Fig. 1b). Out of the 132 known ORFs in the SpliMNPV-AN1956 genome^13^, the 39 cSSRs identified in this study successfully covered 33 (25% of the total ORFs). Assessing the relative composition of the repeat types within the covered ORFs revealed that the 38 cSSRs (97.4%) concerned were predominantly composed of trinucleotide repeats with only one mononucleotide motif (2.6%) identified. Of the 33 ORFs covered by cSSRs, ORF–14 was found to have the highest number of cSSR motifs (3 trinucleotide cSSR motifs).

### Frequency of Classified Repeat Types

Frequency analysis of the classified repeat types revealed that the SpliMNPV-AN1956 genome had seven types of trinucleotide repeats: AAC/GTT, AAT/ATT, ACG/CGT, ACT/AGT, AGC/CTG, ATC/ATG, and CCG/CGG. The CCG/CGG repeats were the most prevalent, whereas the AAT/ATT repeats were the least represented. Mono-, di- and hexanucleotide repeats were found to be composed of only one type of each (A/T, AC/GT and AATACT/AGTATT). The distribution of mono-, di-, tri- and hexanucleotide repeats is summarized in Table 1.

### Development and Characterization of cSSR Markers

**Figure 2:**
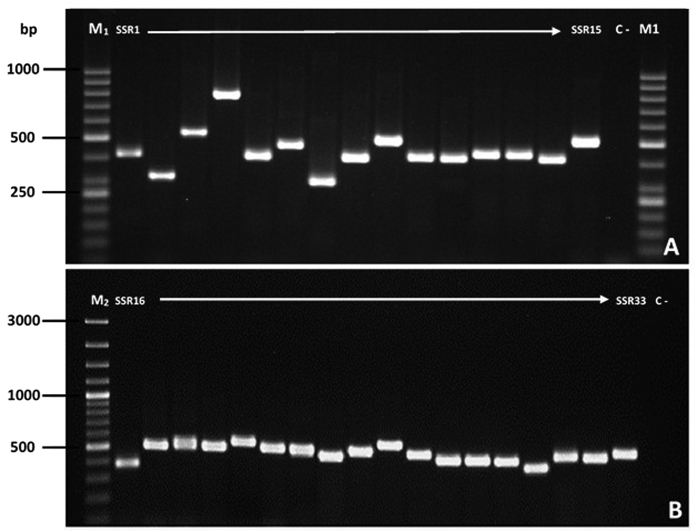
Representative electrophoresis gel showing the PCR amplicon of the developed cSSR markers in SpliMNPV. (**A**) cSSR markers from SSR1 to SSR15 (annotated as defined functional genes). (**B**) cSSR markers from SSR16 to SSR33 (annotated as hypothetical protein). M1: 50 bp DNA ladder, M2: 100 bp ladder plus, C: Negative controls (PCR using DNA extracted from non-infected *S. littoralis* larvae).

Out of the 55 microsatellite motifs that were identified in the SpliMNPV genome, 39 motifs (located in coding regions) were analyzed *in vitro* using PCR analysis. We designed 33 SSR-PCR primer pairs to amplify the 39 motifs. The number of cSSR primers designed were less than the targeted amplified motifs due to the fact that some genes harbored more than one SSR motif. The cSSRs name, motif, motif length, motif position, gene, ORF number and protein identification are summarized in Table 2. From the 55 identified SSR motifs, 39 motifs (cSSRs) were selected and 33 primer pairs were designed in order to generate amplicons containing the targeted motifs (Table 2). The 33 cSSR markers produced reliable and reproducible PCR products with the expected molecular size (Fig. 2).

### Alignment and Mapping of cSSRs

**Figure 3:**
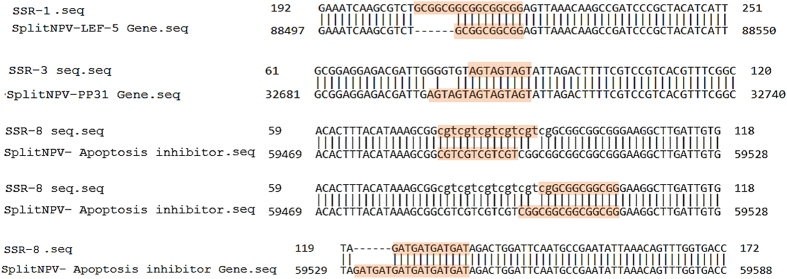
Diagrammatic representation illustrate the differences in SSR motifs between the tested SpliMNPV genome and the published genome.

**Figure 4:**
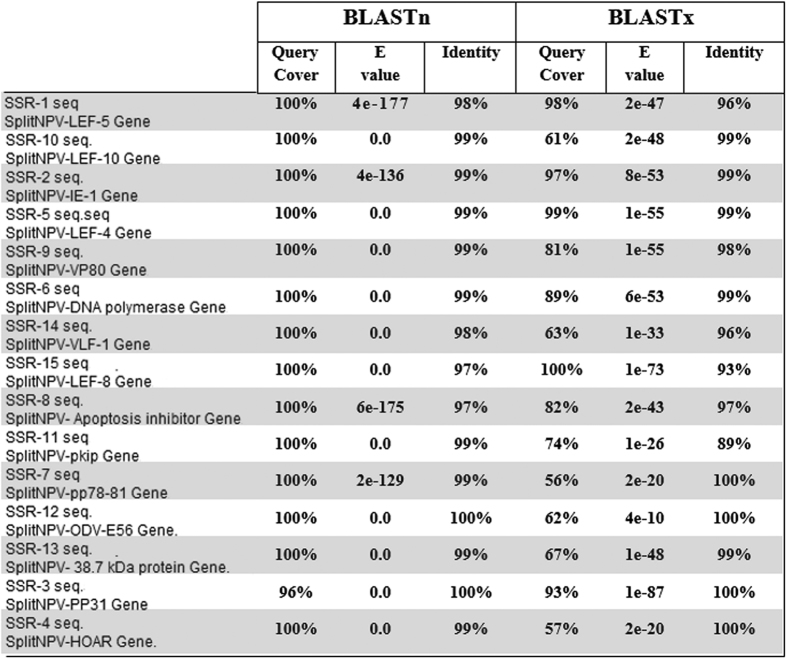
Alignment of the 15 sequenced cSSR markers (partial genes) against their original sequences distributed over the SpliMNPV-AN1956 isolate complete genome.

**Figure 5:**
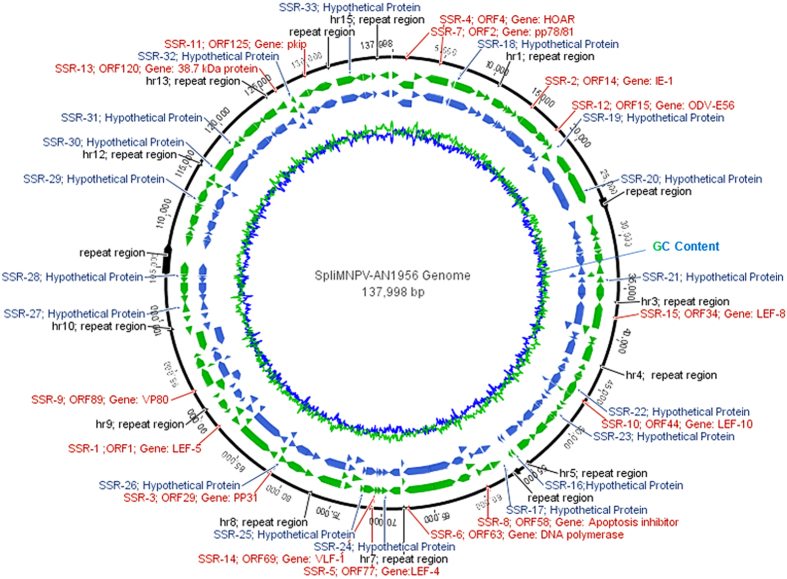
Diagrammatic representation of the SpliMNPV-AN1956 genome showing genes (blue arrows), CDS (green arrows), GC content (blue-green peaks), repeat regions (black arrows), SSRs localized within defined functional gene sequences (SSR–1 to SSR–15; red font), and SSRs localized within CDS sequences annotated as hypothetical protein (SSR–16 to SSR–33; blue font) as localized/distributed on genome.

BLASTn and BLASTx were used to align the 15 cSSR sequences with the GenBank database. The results of BLASTn alignment revealed a high degree of query coverage (96–100%) and a high identity percentage (97–100%) between the 15 cSSR sequences and their equivalent genes from the published SpliMNPV-AN1956 isolate genome sequence. Interestingly, BLASTn alignment of SSR1 and SSR8 in the tested SpliMNPV genome revealed the presence of a novel triplet motif which is not observed in the published SpliMNPV genome sequence. In contrast, the sequence data analysis revealed the absences of three triplet motifs (one in SSR3 and two in SSR8) in the tested SpliMNPV genome when it was compared with the published genome (Fig. 3). The results of BLASTx alignment revealed various degrees of query coverage (57–100%) and a high identity percentage (89−100%) with their equivalent amino acid sequences as derived from the published SpliMNPV-AN1956 annotated genome (Fig. 4). Furthermore, the 33 cSSRs markers were mapped/localized on the SpliMNPV-AN1956 genome. Of 33 cSSRs, 15 were mapped within defined functional genes while 18 have been mapped within CDS sequences annotated as hypothetical proteins (Fig. 5).

## Discussion

With next-gen DNA sequencing technologies becoming increasingly efficient, fast, and cheap, a large number of baculoviral genome sequences are now being generated and made publicly available. These genome sequences represent a potentially valuable resource for mining SSR markers. In the present study, we have identified and characterized 39 cSSRs from a total of 55 SSRs motifs distributed within the SpliMNPV genome (isolate AN–1956). It was observed that the relative abundance of SSR motifs in the SpliMNPV genome (~138 Kb) was 0.39 motif/kb. When compared with the herpes simplex virus type 1 (HSV–1) which has relatively bigger genome size (152 Kb), it was observed that the relative abundance of SSR motifs in the SpliMNPV genome was comparatively lower (0.39 motif/kb for SpliMNPV vs. 0.52 motif/kb for HSV–1). Interestingly, alpha virus, which has small genome size (~11.5 kb), has relative abundance values ranging between 2.32–5.05 motif/kb^14^. These observed variations in the relative abundance of SSR motifs between different viral genome may be attributed to differences in genome sizes or virus type. In the specific case of the SpliMNPV genome, results revealed that the trinucleotide motif was the most abundant type of repeat (80%) followed by the dinucleotide (13%). In partial agreement with our results, dinucleotide and trinucleotide SSRs were reported as the most frequently observed repeat types in the Human Immunodeficiency Virus Type 1 (HIV–1) genomes while tetra-, penta- and hexanucleotide SSRs were almost non-existent^15^. A similar survey of microsatellites in the hepatitis C virus (HCV) revealed that mono-, di- and trinucleotide repeat types were dominant while other types of repeats were observed to occur very rarely^7^. In a sharp contrast, an exploration of 30 alphavirus genomes revealed that mononucleotide repeats were the most prevalent followed by dinucleotide and trinucleotide repeats^14^. A study on the HSV–1 genome reported that mononucleotide repeats occurred with the maximum frequency followed by trinucleotide and dinucleotide repeats^16^. The exploration of microsatellites in diverse Gemini virus genomes showed that among the analyzed genomes dinucleotide repeats were the most abundant followed by the trinucleotide ones; the relative abundance of tetra−, penta−, and hexanucleotide repeats was seen to be very low^17^. Also, a genome wide survey of microsatellite distribution in ssDNA viruses that infect vertebrates revealed that mononucleotide repeats were the most dominant followed by dinucleotide and trinucleotide repeats^18^.

### Comparative distribution across coding and non-coding regions

The distribution of SSRs motifs among coding/non-coding region in the SpliMNPV genome revealed a high incidence (71%) of repeats within coding regions as compared to the non-coding regions (29%). Furthermore, an assessment of the relative composition of repeat motifs revealed that the coding regions predominantly contained trinucleotide repeats (97.4%) with only a solitary mononucleotide repeat sequence (2.6%). In contrast, within non-coding regions it was seen that the di-nucleotide motifs (43.7%) were the most prevalent followed by trinucleotide motifs (37.5%). In agreement with our results, Chen *et al*.^7^ have found that coding regions of the HCV genomes are significantly richer in microsatellite composition as compared to non-coding regions. In *Escherichia coli*, which serve as a prokaryotic model, coding regions are richer in microsatellites as compared to non-coding regions; this can be attributed to the fact that the bulk of the genome is composed of open reading frames^19^,^20^.

### Tri-nucleotide repeats

It was recently reported that coding regions of eukaryotic and prokaryotic genomes have a higher density of trinucleotide repeats as compared to any other repeat type^8^,^21^,^22^. Interestingly, dynamic mutations in trinucleotide repeats have occasionally been associated with the development of some diseases^23^, as well as in other important functions^24^. Microsatellite mutations are more frequently observed in trinucleotide repeats than in any other type of repeats; it is also known that microsatellites can alter their overall length by deletion (contraction) or insertion (expansion) of a small number of repeat units^25^. Interestingly, in the current study, variations between the published genome sequence and the tested SpliMNPV genome have been observed for SSR1, SSR3 and SSR8. These variations can be attributed to contraction of the template strand via loop formation or expansion via replication slippage; the latter is considered as the most likely mutational process in case of the trinucleotide repeats type^26^. In eukaryotes, triplet repeats are more common than non-triplet ones as changes in non-triplet repeats lead to frameshift mutations within coding sequences^8^,^27^. Studies with the alphavirus genomes^14^ have demonstrated that tri-nucleotide repeats are the third most abundant SSRs.

### Di-nucleotide repeats

Dinucleotide repeats are reported to increase the number of expected slippage events per unit length of DNA as they have the highest slippage rate as compared to any other type of repeat^28^. Among 257 viral genomes examined in a published study, it was found that dinucleotide SSRs account for the largest proportion of repeats with the others occurring in significantly lesser proportions^29^. In case of SpliMNPV, dinucleotide repeats were found to be the second most abundant type of repeats following the trinucleotide. It is noteworthy that the exact opposite findings were observed in geminivirus genome. In that case, dinucleotide repeats were significantly more common that the trinucleotides^17^. This can be attributed to the elevated instability rate of dinucleotide repeats due to their higher slippage rate^30^. Additionally, a genome wide survey of microsatellites in ssDNA viruses that infect vertebrates revealed that dinucleotide repeats are the second most frequently occurring type followed by the trinucleotide repeats^18^. Dinucleotide repeats are also speculated to be recombination hot spots^31^,^32^,^33^. This function rapidly adjusts to the evolutionary demands through recovery of genetic variation lost by genetic drift^34^,^35^.

### Mononucleotide repeats

The results obtained in this study clearly demonstrate that mononucleotide repeats have a rare occurrence in the SpliMNPV-AN1956 genome. Poly (A/T) repeats occur more frequently as compared to poly (G/C) repeats (Table 1). The SpliMNPV genome is known to have a relatively high GC content of 44.68% and it is generally assumed that the higher poly (G/C) frequencies in the genome are attributable to the high GC content of the genome^36^. In this context it was interesting to find that poly (G/C) repeats were entirely absent in the SpliMNPV genome. Hence it can be concluded that GC content of genome has negligible or no influence on the occurrence of mononucleotide repeats; this is particularly true for poly (G/C) repeats in the SpliMNPV genome. In general, in eukaryotic or prokaryotic genomes, it has been observed that poly (A/T) tracts are more abundant than poly (G/C) tracts^10^,^20^,^36^,^37^. In the same context, in baculoviruses the frequency of A/T mononucleotide repeats was found to be significantly higher than that of the G/C mononucleotide repeats^38^. In yeast and *E. coli*, mononucleotide repeats were found to strongly affect protein expression by virtue of higher error rates of transcription and translation^36^,^39^,^40^,^41^.

### Microsatellites as a component of viral genomes

In this study, a variety of simple sequence repeats were identified and characterized in the SpliMNPV genome. It was observed that some microsatellite types were significantly over represented which is suggestive of the fact that they may play an important role in SpliMNPV genome organization. In viruses, microsatellites are known as the most hypermutable regions^42^. Mutation rates of SSRs have been reported to be affected by a variety of parameters such as motif composition, motif length, and purity of repetition^27^. The functional and evolutionary role of microsatellites in baculoviruses is poorly understood and further studies are needed in order to explore their distribution and frequency within these genomes. Variations in their complexity and frequency across species and also within coding and non-coding sequences is suggestive of the fact that they may be involved in the recombination process occurring within hot spots and consequently be important from a gene regulation point of view^14^. Also, microsatellites have been reported to be involved in different processes such as replication, recombination, and repair mechanisms, which in turn results in sequence diversity that drives adaptive forces^24^. Some pathogens are found to have the ability to utilize SSRs to frustrate the host immune system by using it to enhance their antigenic variability^29^. It has been reported in literature that errors in high fidelity polymerase activity are not the only reason for any evolutionary event to occur within virus genomes, but that it may also be governed by replication speed and genomic architecture of the virus^43^. In conclusion, the study of microsatellites in SpliMNPV genome is the first step towards a better understanding of the nature, function and evolutionary biology of baculoviruses. Additionally, microsatellites are also known to provide a molecular basis for virus persistence and adaptation to environmental stresses. Our preliminary results can be considered as a useful tool in the study of viral genetic diversity, virus evolution and strain demarcation. Our group is in the process of conducting similar studies on all completely sequenced baculoviral genomes in order to elucidate the functional significance and evolutionary dynamics of microsatellites.

## Methods

### SpliMNPV Genome sequence

The publicly available whole genome sequence of SpliMNPV isolate AN1956 (Accession no. JX454574), as obtained from the NCBI database (http:// http://www.ncbi.nlm.nih.gov/nuccore/449139050), was used for genome-wide *in silico* microsatellites analysis. More information on this genome can be obtained from data published by Breitenbacha *et al*.^13^. Both the genomes, i.e., the publicly available genome used for the *in silico* analysis as well as the isolate used for SSR *in vitro* identification, are Egyptian in origin.

### Genome-wide Microsatellites Identification

The following criteria were used to configure the MIcroSAtellite (MISA) identification tool software to identify SSRs: mono-nucleotide (×10), di- (×6), tri- (×5), tetra- (×5), penta- (×5) and hexa-nucleotide (×5). This tool facilitates the identification and localization of perfect and compound microsatellites. Identified SSRs were classified as coding (cSSRs) and non-coding based upon their presence within coding or non-coding regions of the SpliMNPV genome. The maximum distance permitted between two different SSR in a compound sequence was 100 bp. Subsequently, Primer3Plus web tool (http://www.bioinformatics.nl/cgi-bin/primer3plus/primer3plus.cgi/) was used to design primer pairs flanking each identified SSR motif located within the coding regions.

### Insect and virus

Cotton leafworm, *S. littoralis* (Boised.) used for virus propagation, was obtained from the Insect Rearing Unit (IRU), Agricultural Genetic Engineering Research Institute, Agricultural Research Center, Giza, Egypt. Larvae were reared on a semi-artificial diet comprising of dry beans, yeast, agar and ascorbic acid sterilized for 20 min at 120 °C^44^. The SpliMNPV, a local Egyptian isolate (AN1956), was used in this study. Viral Occluded Bodies (OBs) were propagated by individually orally inoculating each early fourth instar larvae (L4) of *S. littoralis* with 1000 OB/larva on a small piece of medium for 24 h. Five to seven days post infection (p.i), cadavers were collected and subjected to purification of OBs by homogenizing them in 0.5% sodium dodecyl sulfate (SDS). The homogenate was filtered through two layers of cheesecloth and cotton prior to being washed with additional volumes of 0.5% SDS. Suspended OBs were collected after centrifugation at 12,000 rpm for 10 min. Pellets were washed twice with 0.1% SDS and once with 0.5 M NaCl. OBs were resuspended in deionized distilled H_2_O and viral genomic DNA was extracted as described previously by O’Reilly^45^.

### Microsatellite PCR Analysis and Sequencing

Motifs located within defined functional gene sequences were PCR amplified using especially designed SSR-PCR primer pairs. All PCR reactions were performed in 25 μL reaction mixtures containing 1X PCR buffer, 1.5 mM MgCl_2_, 0.2 μM of each deoxynucleoside triphosphate (dNTPs), 1 μM of forward and reverse primers, 1 U of proofreading Taq polymerase (Platinum, Invitrogen) and 25 ng SpliMNPV genomic DNA. PCR amplification was performed in a Thermal Cycler system 2720 (Applied Biosystems, Inc.). The thermo-cycling profile used was as follows: 1 cycle of denaturation (2 min at 94 °C), 35 cycles (30 s at 94 °C, 30 s at Tm of primer, 60 s at 68 °C), and a final elongation step (10 min at 68 °C). The amplified products were resolved by electrophoresis in a 2% agarose gel at 100 volts. Ethidium bromide was used for detection of amplified DNA. The PCR amplified SSR products were visualized and photographed using a Gel Doc™ XR+ System with Image Lab™ Software (Bio-Rad^®^). Subsequently, the PCR products were purified using QIAquick^®^ PCR Purification Kit (QIAGEN, Santa Clarita, CA) and the purified fragments were subjected to nucleotide sequencing using 3100 ABI sequencer (Applied Biosystems, Inc.) as described by Sanger *et al*.^46^. All sequences obtained were analyzed twice in each direction.

### Sequencing Data Analysis

The nucleotide sequence data of the developed SSR markers was aligned against their equivalent genes sequences in the SpliMNPV genome using the MegAlign tool (DNASTAR, Inc.) in accordance with the ClustalW multiple sequence alignment algorithm^47^. In addition, alignment (MegaBLAST, discontiguous-MegaBLAST) analysis^48^ was used to identify specific regions among the reads that may not be well aligned with the SpliMNPV genome. Furthermore, the sequences were also subjected to the BLASTx analysis which compares translational products of the nucleotide query sequence to protein databases (http://www.ncbi.nlm.nih.gov).

## Additional Information

**How to cite this article**: Atia, M. A. M. *et al*. Genome-wide *In Silico* Analysis, Characterization and Identification of Microsatellites in Spodoptera littoralis Multiple nucleopolyhedrovirus (SpliMNPV). *Sci. Rep.* **6**, 33741; doi: 10.1038/srep33741 (2016).

## Figures and Tables

**Table 1:** Characteristics of the repeat types (motifs and their sequence complementary) for the *Spodoptera littoralis* nucleopolyhedrovirus.

Type	Motif	Motif repeats								Total no. of SSR	Frequency %
		5	6	7	8	9	10	15	16		
Mono	A/T						3			3	100.0*
Di	AC/GT		4			1		1	1	7	100.0**
Tri	AAC/GTT	1			1					2	4.5***
	AAT/ATT	1								1	2.3***
	ACG/CGT	4	3	1	2	2				12	27.3***
	ACT/AGT	1	1	1	1					4	9.1***
	AGC/CTG	1	2	1	1					5	11.4***
	ATC/ATG	3	2	2						7	15.9***
	CCG/CGG	8	3	2						13	29.5***
Hexa	AATACT/AGTATT	1								1	100.0****
Total										55	

*Frequency of motifs in total number of mononucleotide SSRs.

**Frequency of motifs in total number of dinucleotide SSRs.

***Frequency of motifs in total number of trinucleotide SSRs.

****Frequency of motifs in total number of hexanucleotide SSRs.

**Table 2:** Characteristics of the 33 microsatellite markers developed for the *Spodoptera littoralis* nucleopolyhedrovirus.

Name	(Motif) length	Position	Gene	ORF	Expected Size (bp)
SSR1	(A)10, (GCG)5*	88474–88483, --------------	LEF–5	ORF84	386
SSR2	(AAC)5	14862–14876	IE–1	ORF14	316
SSR3	(AGT)3**, (TGC)5	32697–32711, 33052–33066	PP31	ORF29	537
SSR4	(TCA)5, (GCG)5, (ATC)7	4215–4229, 4414–4428, 4856–4876	HOAR	ORF4	810
SSR5	(CGA)8	79413–79436	LEF–4	ORF77	409
SSR6	(CGA)5	67543–67557	DNA polymerase	ORF63	464
SSR7	(CGG)5, (GGC)6	1539–1553, 1570–1587	pp78/81	ORF2	301
SSR8	(CGG)5**, (GAT)6**, (CGT)5*	59499–59513, 59531–59548, ---------------	Apoptosis inhibitor	ORF58	414
SSR9	(GCC)5, (GAC)8	92590–92604, 92648–92671	VP80	ORF89	503
SSR10	(GAC)5	46842–46856	LEF–10	ORF44	418
SSR11	(GCA)7	129403–129423	pkip	ORF125	417
SSR12	(GGC)5	18102–18116	ODV-E56	ORF15	439
SSR13	(GTC)7	126169–126189	38.7 kDa protein	ORF120	438
SSR14	(TCG)9	70961–70987	VLF–1	ORF69	413
SSR15	(TCG)6	38044–38061	LEF–8	ORF34	501
SSR16	(TCG)5	55421–55435	HP	hr5	376
SSR17	(TTA)5	57045–57059	HP	hr6	494
SSR18	(TGC)8	7005–7028	HP	ORF6	500
SSR19	(CGA)6	19460–19477	HP	ORF18	500
SSR20	(ATC)6	24789–24806	HP	ORF21	539
SSR21	(GTT)8	34341–34364	HP	ORF31	500
SSR22	(TAG)8	46038–46061	HP	ORF42	469
SSR23	(CCG)7	49243–49263	HP	ORF48	412
SSR24	(TCA)7	69808–69828	HP	ORF66	500
SSR25	(GCC)5	72288–72302	HP	ORF70	556
SSR26	(CAG)6	81544–81561	HP	ORF79	491
SSR27	(ATG)5	101095–101109	HP	ORF96	446
SSR28	(TGC)6	104359–104376	HP	ORF100	442
SSR29	(CCG)6	112560–112577	HP	ORF108	439
SSR30	(GGC)6	116277–116294	HP	ORF113	400
SSR31	(GAC)9	119289–119315	HP	ORF114	456
SSR32	(CGG)7	127202–127222	HP	ORF122	448
SSR33	(TCG)5	133751–133765	HP	ORF128	481

*New SSR motifs not observed in the SpliMNPV published genome sequence.

**Absent SSR motifs in the tested SpliMNPV genome in comparison with the published genome.

HP: Hypothetical protein.
